# A Mixed-Methods Study Using a Nonclinical Sample to Measure Feasibility of Ostrich Community: A Web-Based Cognitive Behavioral Therapy Program for Individuals With Debt and Associated Stress

**DOI:** 10.2196/mental.6809

**Published:** 2017-04-10

**Authors:** Dawn Smail, Sarah Elison, Linda Dubrow-Marshall, Catherine Thompson

**Affiliations:** ^1^ Ostrich Community Manchester United Kingdom; ^2^ Breaking Free Group Manchester United Kingdom; ^3^ School of Health Sciences University of Salford Salford United Kingdom

**Keywords:** cognitive behavioral therapy, computer-assisted therapy, psychological stress, economic recession, mental health

## Abstract

**Background:**

There are increasing concerns about the health and well-being of individuals facing financial troubles. For instance, in the United Kingdom, the relationship between debt and mental health difficulties is becoming more evident due to the economic downturn and welfare reform. Access to debt counseling services is limited and individuals may be reluctant to access services due to stigma. In addition, most of these services may not be appropriately resourced to address the psychological impact of debt. This study describes outcomes from an Internet-based cognitive behavioral therapy (ICBT) program, Ostrich Community (OC), which was developed to provide support to those struggling with debt and associated psychological distress.

**Objective:**

The aim of this feasibility study was to assess the suitability and acceptability of the OC program in a nonclinical sample and examine mental health and well-being outcomes from using the program.

**Methods:**

A total of 15 participants (who were not suffering from severe financial difficulty) were assisted in working through the 8-week ICBT program. Participants rated usability and satisfaction with the program, and after completion 7 participants took part in a semistructured interview to provide further feedback. Before the first session and after the final session all participants completed questionnaires to measure well-being and levels of depression, stress, and anxiety and pre- and postscores were compared.

**Results:**

Satisfaction was high and themes emerging from the interviews indicate that the program has the potential to promote effective financial behaviors and improve financial and global psychosocial well-being. When postcompletion scores were compared with those taken before the program, significant improvements were identified on psychometric measures of well-being, stress, and anxiety.

**Conclusions:**

The OC program is the first ICBT program that targets poor mental health associated with financial difficulty. This feasibility study indicates that OC may be an effective intervention for increasing financial resilience, supporting individuals to become financially independent, and promoting positive financial and global well-being. Further work with individuals suffering from debt and associated emotional difficulties will help to examine clinical effectiveness more closely.

## Introduction

### Internet-Based Cognitive Behavioral Therapy

Cognitive behavioral therapy (CBT) [1,2] is an effective treatment for depression and anxiety recommended by the National Institute for Health and Care Excellence (NICE). It is based on the assumption that most behavioral and emotional reactions are learnt and focuses on the interrelated components of emotions, behaviors, and thoughts [3]. A key advantage of CBT is that it adapts to computerization [4], with Internet-based cognitive behavioral therapy (ICBT) programs tailored to the needs of the individual. There are a range of ICBT programs adapted for different health issues, psychological disorders, and lifestyle choices such as substance misuse [5-8], insomnia [9,10], mild to moderate depression [11,12], pathological gambling [13], and perfectionist-related issues [14]. This feasibility study explores the acceptability and usability of an ICBT program that has been designed for people experiencing mental health problems due to difficult financial circumstances.

In the midst of a recession and with the continuing move by the UK government to implement austerity measures, vulnerable members of society are suffering the consequences of an economic contraction [15]. One of the adverse outcomes of the recession is the so-called “credit crunch” whereby individuals are struggling to cope with debt and the consequences of this on mental health and well-being [16-19]. Individuals in financial distress (especially those experiencing unemployment and impoverishment) manifest a range of illnesses including depression, anxiety, and alcohol use disorders [20,21], with greater austerity measures increasing the severity of such mental health issues [22,23]. Indeed, a survey by Rethink [24] revealed that almost 9 out of 10 of those in debt felt that their financial difficulties had made their mental health problems worse.

### Mental Health Problems and Debt

A further negative consequence of the imposed austerity measures is the continuing erosion of access to mental health services [24,25]. Financial institutions, such as the Citizens Advice Bureau, are not able to support those experiencing financial difficulties and associated mental health problems [26]. Due to limited access to appropriate support and intervention, individuals may therefore be less able to resolve their financial problems or access help, and subsequently, may find themselves sinking deeper into debt.

In light of the detrimental effects that debt may have on well-being, and the lack of evidence-based services that offer both financial and emotional advice, government and financial institutions now recognize that considerable work needs to be done to address this gap in services [27,28]. However, although the optimal treatment for debt and mental health is still being sought, there is good reason to assume that ICBT can be adapted to the specific issues associated with the burden of debt. ICBT may be appealing in that it may reduce concerns an individual has about stigma associated with seeking face-to-face help for debt and mental health issues [29]. Indeed, most of those who experience financial difficulty also experience feelings of shame, self-disgust, and secretiveness [30]. This means that individuals in debt can suffer in silence when confidential services that assure anonymity are lacking [31].

Therefore, this study was conducted to assess the usability and acceptability of a novel ICBT treatment program, Ostrich Community (OC), which is designed to help individuals with their financial difficulties while supporting them to overcome any associated mental health problems. The program aims to promote development of positive behaviors, increase financial knowledge, and improve self-efficacy with regards to financial management. The study adopted a mixed-methods approach as recommended by the Medical Research Council (MRC) guidance around the development and evaluation of complex, multicomponent psychosocial interventions [32,33]. This MRC guidance recommends that before examining clinical effectiveness of complex interventions via approaches such as randomized controlled trials (RCTs), feasibility and piloting work should be conducted in order to ascertain initial acceptability and usability of any novel intervention. This may be particularly important when developing digital interventions that can be perceived as “disruptive” to the status quo within an existing health or social care system [34,35].

In line with the MRC guidance, it was important to conduct a feasibility study to explore acceptability and usability in a group of participants experiencing only moderate financial difficulties, before examining effectiveness in the target population, that is, service users accessing support for financial and mental health difficulties. This approach is important to gain insights into improvements that could be made to the program before evaluating it more formally with a group who may have more complex needs [36]. Small-scale feasibility studies can also reveal insights into acceptability of novel treatment approaches, which can be used to facilitate engagement during formal piloting and effectiveness studies [37-40].

## Methods

### Design

This was a mixed-methods feasibility study, incorporating semistructured qualitative interviews and quantitative satisfaction measures, alongside psychometric measures of stress and well-being. These measures were taken to explore acceptability and usability of the OC program.

### Description of the OC Program

The OC ICBT program provides practical advice about management of finances and psychological well-being and clinically validated evidence-based psychosocial intervention strategies that are grounded in the theoretical underpinnings of the CBT model [3]. Each module provides psychoeducation, cognitive-behavioral worksheets or strategies, and money management stories and quotes to help increase motivation. The program moves beyond information-giving and is the very first intervention program that is evidence-based and tailored to address issues surrounding financial and associated psychological distress.

The program comprises a 2-min introductory video, followed by 8 Web-based sessions at weekly intervals. Each session lasts approximately 30 min, with approximately 30 additional min per week spent practicing away from the computer (eg, keeping problem diaries, thought records, and completing behavioral experiments) in order to encourage the user to practice the skills taught throughout the program. The program contains information delivered by video and audio to enhance engagement and accessibility. See Table 1 for more detail of the content of individual modules and topics covered by the program.

The OC program is best suited to users who are experiencing mild to moderate stress or depression and anxiety symptoms, and who need information and guidance about money management. Participants with more serious mental health symptoms and debt issues are introduced to content on the home page and there is a toolbox that provides information on more intensive mental health and debt advice services that are more appropriate to their needs.

**Table 1:** Content of the Ostrich Community (OC) program.

Module	Topic	Summary of activities
1	What are stress, fear, and anxiety?	Provides information to give awareness and understanding of stress and how it can be prevented and managed. The module introduces the purpose of the program (ie, to support users to develop skills to cope with financial stress), and helps the user to understand the links between financial difficulties and stress (Figure 1).
2	The Ostrich model— therapeutic interventions	Introduces cognitive behavioral therapy and self-help techniques to help the user understand why they feel as they do, and learn strategies for changing the way they think, feel, and act. It uses videos, text, and exercises that focus on generic knowledge and skills to help the individual apply these methods in their own lives, with a specific focus on applying these skills to coping with difficult financial situations.
3	Thinking patterns	Provides information, videos, and exercises to help the user identify and challenge negative thoughts and learn to think in a more accurate and realistic way toward their financial situation (Figure 2). Uses cognitive restructuring and other cognitive change techniques to encourage accurate appraisal of financial difficulties.
4	Problem solving	Builds on the cognitive techniques provided in session 3 to support users to utilize their new, realistic insights into their financial situation to solve identified difficulties. The problem solving approaches are underpinned by a goal-setting strategy, to encourage the user to set realistic, achievable goals. Mind mapping exercises are used to identify possible solutions to barriers to goal attainment. Promotes help seeking behaviors (seeking debt advice, talking to creditors).
5	Incorporating positive actions	Helps to identify unhelpful and unhealthy behaviors, particularly around finances, and provides guidance on changing these into healthier behaviors. Provides techniques to increase coping skills and effective communication skills to assist the user in talking to other agencies about their debt, for example. This session also includes completion of an activity monitoring and scheduling planner, to enable the user to organize days and times that they will carry out problem-solving activities related to their debt.
6	Taking action	Provides practical information and resources directly related to financial issues, such as budgeting exercises, and guides the user through a step-by-step exercise to become more financially competent.
7	Stress management and relaxation methods	Provides more general exercises, information, and videos of wider stress management techniques, including relaxation training, progressive muscle relaxation, visualization, and gentle exercise, to help promote relaxation and enhance ability to cope with financial stress.
8	Recap	This module recaps on prior learning and information covered in the program to help consolidate and reinforce learning and the practical skills taught.

**Figure 1:**
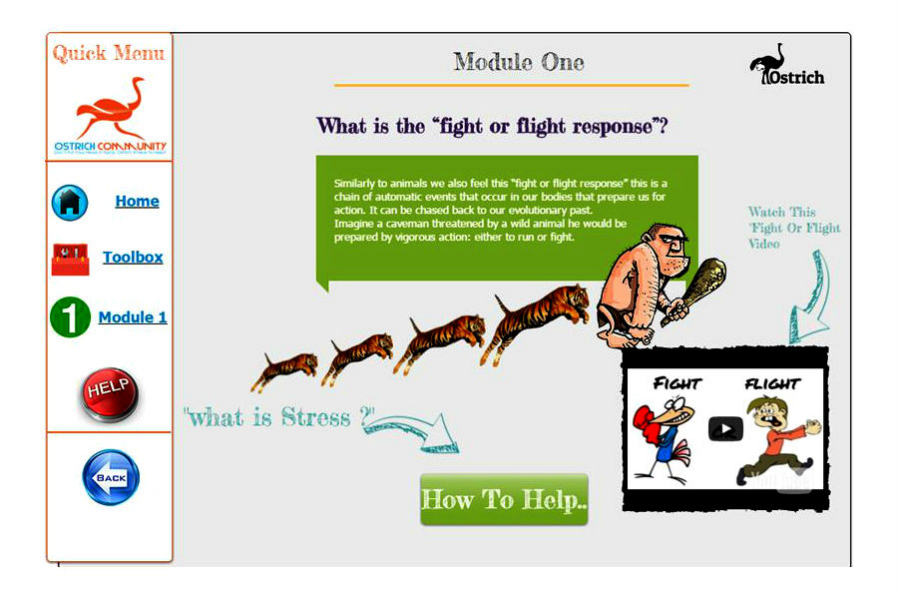
A screenshot from Module 1 that focuses on educating the user about stress and anxiety.

**Figure 2:**
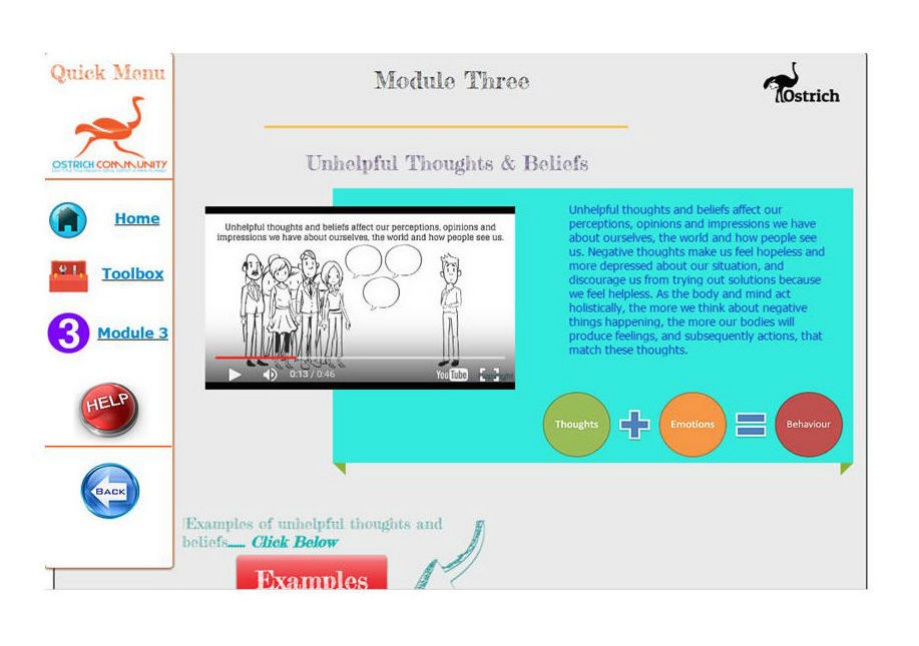
A screenshot from Module 3. This activity encourages users to challenge their beliefs and thought patterns.

### Participants

The sample consisted of 17 students from the University of Salford, who were not in significant financial distress, but were coping with the demands of a further education course of study, alongside the low income and financial difficulties many students experience. This group was selected as it was considered important to explore acceptability and usability of the program in a group without significant financial and emotional difficulties, before testing individuals with more complex needs that are facing real financial and emotional difficulties. All participants were studying full-time and some were also working to supplement their income. Table 2 provides demographic information on the sample.

**Table 2:** Participant demographics for those who volunteered for the program (N=17).

Demographic characteristics		n (%)
**Sex**		
	Female	11 (65)
	Male	6 (35)
**Age range (years)**		
	18-24	7 (41)
	25-34	8 (47)
	35-44	2 (12)
**Marital status**		
	Single	14 (82)
	Married	2 (12)
	Separated	1 (6)
**Employment status (alongside being a student)**		
	Working (full-time)	4 (24)
	Working (part-time)	3 (18)
	Student only (no full- or part-time work)	10 (59)
**Living arrangements**		
	Home owner	3 (18)
	Renting a property	9 (53)
	Living with parents (not paying rent)	5 (29)

### Procedure

Participants who consented to take part subscribed to the program by activating a Web-based account, providing an email address, and creating a password. They received a welcome message together with login details via email. Of the 17 consenting participants, 15 activated an account.

**Figure 3:**
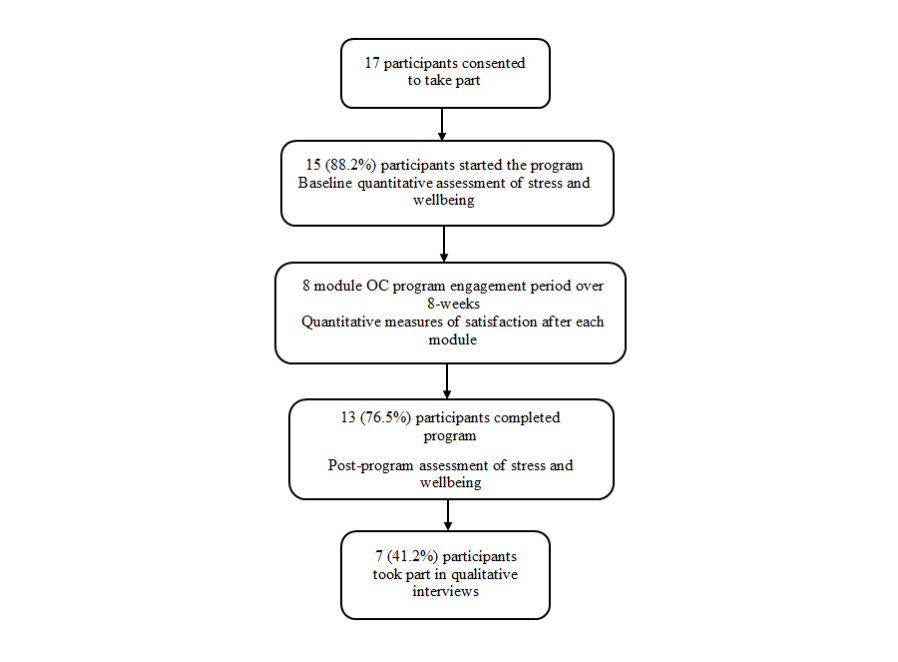
Flowchart depicting study stages and participant attrition.

Participants worked through the modules sequentially, with a new module released each week for 8 weeks together with an email reminder. Following completion of all modules, participants were invited to take part in a semistructured interview exploring acceptability and usability of the program (7 took part). Before module 1 and after module 8, participants completed two standardized psychometric assessments, measuring stress and well-being (with a total of 13 participants providing these data). The flowchart depicted in Figure 3 demonstrates the sequence of events during the study, from initial recruitment to completion of qualitative interviews, and shows attrition rates at each stage.

### Measures

Three separate methods were used to examine (1) acceptability and usability of the program, (2) participant satisfaction, and (3) stress and well-being outcomes.

#### Feasibility Study: Qualitative Interviews to Assess Acceptability and Usability

On completion of the program, participants were offered the opportunity to take part in an interview. Interviews were conducted on a one-to-one basis with the lead author (DS) and explored participants’ views of the OC program. Open-ended questions allowed participants to elaborate on their answers. Interviews lasted approximately 30 min and were recorded using a digital Dictaphone.

The interviews were transcribed and entered into NVivo qualitative data analysis software version 10 (QSR International Pty, Ltd), and thematic analysis (TA) was used to analyze the transcripts. Published guidelines around the process of TA in psychological research were followed [41], with transcripts initially being read by the lead author (DS) to identify quotes relevant to the study aims. Connections were made between quotes and were used to develop overarching themes, which were examined and refined to ensure that they accurately described themes common to all transcripts. These were reviewed by the second author (SE), to increase reliability, and any discrepancies were discussed until an agreement was reached. The themes were named and defined to ensure that they were discrete.

#### Feasibility Study: Quantitative Measures to Assess Satisfaction

This stage of the study measured satisfaction with the program using the 8-item Client Satisfaction Questionnaire (CSQ-8) [42]. The CSQ-8 total score ranges from 8 to 32, with higher scores indicating higher satisfaction. Participants completed this at the end of every session and at the end of the program. A short questionnaire developed by the lead author was also used to gain additional information around satisfaction and usability specific to the OC program, and this was completed after each module. This was a 5-item questionnaire with a Likert scale ranging from 0 (“not at all”) to 7 (“very much”) and participants were asked to state how much they agreed with statements such as “How useful did you find the new topic introduced today?” Data were analyzed using Microsoft Excel and Statistical Package for the Social Sciences (SPSS).

#### Clinical Outcomes: Quantitative Psychometric Measures of Stress and Well-Being

Participants completed the Warwick and Edinburgh Mental Well-Being Scale (WEMWBS) [43] and the short version of the Depression Anxiety Stress Scale (DASS-21) [44] to monitor any changes in well-being and stress as a result of engaging with the program. The WEMWBS is a scale of 14 positively worded items for assessing mental well-being. Each statement asks for a response on a Likert scale that ranges from 1 (“None of the time”) to 5 (“All of the time”). The scores on the questionnaire range from 14 to 70 with higher scores indicating greater well-being. The DASS is a 21-item self-report questionnaire designed to measure the severity of a range of symptoms common to depression, anxiety, and stress with 7 items for each scale. Each item is scored on a 4-point scale from 0 (“Did not apply to me at all”) to 3 (“Applied to me very much or most of the time”) indicating how often the respondent experienced each state in the past week. The questionnaire provides 3 scores (one for each state) with higher scores indicating that respondents had suffered from a particular state in the last week.

In order to determine stress and well-being outcomes, pre- and postintervention scores on the DASS [44] and WEMWBS [43] were subjected to statistical analyses. Nonparametric Wilcoxon Signed-Ranks tests were used to examine changes in scores at the beginning and end of the program.

## Results

As three methods were used to address three independent aims, findings from each of these are reported separately.

### Feasibility Study: Qualitative Interviews to Assess Acceptability and Usability

Three themes were identified relating to (1) enhancing engagement with the OC program, (2) acceptability of program content, and (3) the programs’ potential to benefit the target population.

#### Theme 1: Enhancing Engagement

Participants reported a number of features of the program that enhanced engagement, including presentation of modules in a sequential and progressive manner with each one building on knowledge gained from the previous session:

it was organized in a way that you can do it, in a step-by-step process.

Participant 1

Participants also liked that the program content was delivered via interactive Web-based multimedia including video and audio, although one participant did suggest that a purely Web-based program may not be suitable for everyone:

I think...the way the website was very visual, and you did a blend of different ways (to) suit people’s needs in terms of their learning style (but) I think it needs to be maybe classroom based...there’s a tutor or someone taking them through it bit by bit then taking them back to apply it.

Participant 6

#### Theme 2: Acceptability and Accessibility of Content

In general, participants were positive about the content of the program, and while there was acknowledgment from participants that the content was informed by academic theory, this did not make it difficult to access or understand:

I liked that there was a good balance between the theory, the information provided, and activities...it was nothing too strenuous.

Participant 7

Positive reinforcement within the program was also perceived as helpful and played an important part in supporting users to achieve the goals they set:

I like the positive quotes and encouraging words that were throughout the program, I liked how rewarding it was...which made you feel like you’ve done some progress.

Participant 4

#### Theme 3: Potential to Benefit the Target Population

On the whole, participants reported that they understood the concept of the program, even though a Web-based financial capability program was a new idea:

it was a new concept that I haven’t explored in other programs. It’s a financial capability program so its specialized and it’s good that its specialized to help people with financial difficulties in that sense.

Participant 1

The opinion among participants was that the program offered practical advice and solutions to financial difficulties and would support individuals to develop the tools necessary to overcome their financial difficulties:

it was practical, there was so many things you provided in the tool box, which is a good thing because the program didn’t just talk about stuff it actually provided the tools to help people.

Participant 3

### Feasibility Study: Quantitative Measures to Assess Satisfaction

All 13 participants completed the usability questionnaire after each module and the CSQ-8 [42] at the end of the program. Data from the usability questionnaire (Table 3) demonstrated mean experience across the 8 sessions was favorable, with participants rating the usefulness of each topic and the benefits of each session as high. Following each session participants felt less stressed compared with how they felt at the beginning, with none reporting distress from any of the sessions.

**Table 3:** Perception of the sessions based on scores from the usability questionnaire completed after each module (N=13).

Questions	Mean (SD^a^)
How stressed were you at the beginning of the session?	3.63 (0.74)
How useful did you find the new topic introduced today?	6.13 (0.64)
How relaxed do you feel right now?	5.88 (0.35)
How beneficial did you find the session?	6 (0.53)
How tense do you feel right now?	2.25 (0.46)

^a^SD: standard deviation.

From the CSQ-8 data (Table 4), 92% (12/13) of participants rated the quality as good or excellent. All participants received the service they wanted, and 92% (12/13) reported that the program met most or all of their needs and that they were satisfied with the amount of help provided. All would recommend the program to a friend in need of financial help and 92% (12/13) said that they would come back to the program if they needed help in the future. When asked how satisfied they were with the program overall, 69% (9/13) were “very satisfied” and 30% (4/13) were “mostly satisfied.”

**Table 4:** Satisfaction with the overall program based on the 8-item Client Satisfaction Questionnaire (CSQ-8; N=13).

Questions		n (%)
How would you rate the quality of service you have received?	Excellent	6 (46)
	Good	6 (46)
	Fair	1 (8)
	Poor	0 (0)
Did you get the kind of service you wanted?	Yes, definitely	8 (62)
	Yes, generally	5 (38)
	No, not really	0 (0)
	No, definitely not	0 (0)
To what extent has the program met your needs?	Almost all of my needs have been met	7 (54)
	Most of my needs have been met	5 (38)
	Only a few of my needs have been met	1 (8)
	None of my needs have been met	0 (0)
If a friend were in need of similar help would you recommend the program?	Yes, definitely	9 (69)
	Yes, I think so	4 (31)
	No, I don’t think so	0 (0)
	No, definitely not	0 (0)
How satisfied are you with the amount of help you have received?	Very satisfied	11 (84)
	Mostly satisfied	1 (8)
	Indifferent or mildly dissatisfied	1 (8)
	Quite dissatisfied	0 (0)
Have the services you have received helped you to deal more effectively with your problems?	Yes, they helped a great deal	6 (46)
	Yes, they helped	6 (46)
	No, they seemed to make things worse	0 (0)
	No, they really did not	1 (8)
In an overall, general sense, how satisfied are you with the service you have received?	Very satisfied	9 (69)
	Mostly satisfied	4 (31)
	Indifferent or mildly dissatisfied	0 (0)
	Quite dissatisfied	0 (0)
If you were to seek help in the future would you come back to the program?	Yes, definitely	8 (62)
	Yes, I think so	4 (31)
	No, I do not think so	1 (8)
	No, definitely not	0 (0)

### Clinical Outcomes: Quantitative Measures of Stress and Well-Being

Participants reported higher levels of anxiety before the program (median 24) than at the end (median 16) and this difference was significant, *Z* (13)=−2.006, *P*<.05 There was also a significant difference in stress scores before and after (median scores of 32 and 26 respectively) with participants reporting lower levels of stress at the end of the program, *Z* (13)=−2.814, *P*<.01. There was no difference between levels of depression at the end of the program (median 22) compared with the beginning (median 26), *Z* (13)=−0.393, *P*=.69.

Scores on the WEMWBS were lower at the beginning of the program (median 45.00) compared with the end (median 51). Participants reported a significant increase in well-being from pre- to postprogram, *Z* (13)=−2.275, *P*<.05.

## Discussion

### Principal Findings

The continuing government austerity measures, coupled with a bleaker outlook on jobs, have resulted in more people experiencing debt [15]. This often takes a toll on a person’s health and psychological well-being [16] and is exacerbated by a shortage of services available to assist people experiencing financial hardship and associated mental health difficulties. Financial and other governmental institutions have limited resources to assist people in this situation and the waiting list of people needing help is expanding [45].

The OC program is an innovative ICBT program that aims to support individuals who are having financial difficulties that are affecting their emotional well-being. The focus of the program is to relieve psychological stress, anxiety, and depression while teaching positive financial behaviors and resilience. This initial feasibility study was conducted to explore acceptability and usability of the program.

Many participants said that they appreciated taking part in the study because it helped them to learn how to budget effectively and how to be prepared in terms of dealing with sudden financial changes. One of the benefits of this program is therefore that individuals can use it as a preventative tool regardless of their financial circumstances and in the absence of significant levels of debt [2,46]. Indeed, participants noted that the CBT principles in the program could be beneficial for anyone, even for those without any significant financial or psychological difficulties. In addition, after completing the program, participants reported a significant reduction in anxiety and stress, and improvements in well-being.

Participants found the program to be helpful and easy to use and there was high satisfaction with the financial information and emotional help provided. These findings support the high satisfaction with ICBT when addressing gambling [13], insomnia [10], mild-to-moderate depression [11], and drug and alcohol use [6,8]. Participants liked the interactive and multimedia, digital format of the program, reporting that they found it to be user-friendly, and the information easy to understand. Some observed that although the program allows for people to work independently, it may be too much for some who may benefit from “computer-assisted therapy” (CAT) rather than “self-help therapy.”

CAT integrates therapeutic content delivered by an ICBT program with a human practitioner who may provide screening, supervision, and other support to the user [477], whereas self-help programs do not use practitioner monitoring or involvement [48]. Evidence suggests that people with higher levels of motivation, such as students, have improved outcomes from participation in this latter type of program. Students are generally driven by a desire to achieve [49] and are encouraged to be self-motivated autonomous learners [50]. This is a drawback with this sample as participants may have been more motivated than those in the general population suffering with debt problems. This is supported by the relatively low dropout rate compared with other studies of ICBT programs, some of which report high attrition rates [51]. In addition, as the students who participated in this study were not necessarily in debt, and instead were coping with a relatively low income that is common among students, the sample is not representative of the target user groups.

A “blended” approach to facilitation of ICBT programs has been demonstrated to be helpful for those individuals experiencing mental health difficulties [52]. Therefore, further work will explore delivery of the program not as self-help, but as CAT. By delivering the program as CAT with practitioner support, this may lessen the attrition rate in the target population, and may provide vital additional therapeutic support for individuals experiencing significant financial hardship and mental health difficulties [51].

This study intended to explore initial feasibility and acceptability of the OC program with a small cohort of participants experiencing some financial difficulties. This was to gain insights into improvements that could be made to the program before evaluating it with a population of service users with more complex needs. Multiple digital health studies take this approach, including studies of mental health intervention programs [53] and alcohol misuse programs [36]. The sample size was limited, yet when examining feasibility of novel digital interventions such as the OC program, small-scale studies can reveal important insights regarding acceptability and clinical content. This can facilitate optimal engagement during formal piloting and effectiveness studies [37-40]. Future work will include a larger pilot study with the target population, that is, service users accessing support for their financial and mental health difficulties.

### Conclusions

The aims of this feasibility study were to assess the acceptability and usability of a novel ICBT program for people experiencing poor mental health due to financial difficulties. A total of 15 participants worked through the program over a period of 8 weeks, and they reported satisfaction with the program and noted that it was easy to use. They also suggested that it made them consider their financial situation and gave them skills they could use beyond the immediate focus of finances. There was also a significant improvement in well-being, anxiety, and stress on completion of the program.

Some participants reported that it was difficult to work through the modules individually and so future work will explore the addition of support from a practitioner and potentially a moderated peer support forum. These implementation approaches may help to increase impact [51], increase engagement, and support completion. The sample was also limited to participants not in serious financial difficulties. Therefore, further research will include a larger sample of participants who come from the intended targeted population for the OC program; individuals experiencing significant financial difficulties and associated mental health difficulties.

## Abbreviations

CAT: computer-assisted therapy
CBT: cognitive behavioral therapy
CSQ: Client Satisfaction Questionnaire
DASS: Depression Anxiety Stress Scale
ICBT: Internet-based cognitive behavioral therapy
MRC: Medical Research Council
NHS: National Health Service
NICE: National Institute for Health and Care Excellence
OC: Ostrich Community
RCT: randomized controlled trial
SPSS: Statistical Package for the Social Sciences
TA: thematic analysis
WEMWBS: Warwick and Edinburgh Mental Well-Being Scale
